# A Strategy Formulation Framework for Efficient Screening during the Early Stage of a Pandemic

**DOI:** 10.3390/tropicalmed8020078

**Published:** 2023-01-21

**Authors:** Shuangyan Wang, Yuan Zhang, Qiang Zhang, Qibin Lu, Chengcheng Liu, Fangxin Yi

**Affiliations:** 1 Party School of the Central Committee of C.P.C. (National Academy of Governance), Beijing 100089, China; 2 School of Social Development and Public Policy, Beijing Normal University, Beijing 100875, China

**Keywords:** viral pandemic, screening strategy, scenario model, public health

## Abstract

For viruses that can be transmitted by contacts of people, efficiently screening infected individuals is beneficial for controlling outbreaks rapidly and avoiding widespread diffusion, especially during the early stage of a pandemic. The process of virus transmission can be described as virus diffusion in complex networks such as trajectory networks. We propose a strategy formulation framework (SFF) for generating various screening strategies to identify influential nodes in networks. We propose two types of metrics to measure the nodes’ influence and three types of screening modes. Then, we can obtain six combinations, i.e., six strategies. To verify the efficiencies of the strategies, we build a scenario model based on the multi-agent modelling. In this model, people can move according to their self-decisions, and a virtual trajectory network is generated by their contacts. We found that (1) screening people will have a better performance based on their contact paths if there is no confirmed case yet, and (2) if the first confirmed case has been discovered, it is better to screen people sequentially by their influences. The proposed SFF and strategies can provide support for decision makers, and the proposed scenario model can be applied to simulate and forecast the virus-diffusion process.

## 1. Introduction

During the early stage of a pandemic, a new virus could be spreading quickly because of unprecedented events, limited knowledge of the virus, the lack of a vaccine or specific medicine, and limited resources [1]. In a situation with limited knowledge of a virus, discovering all infected cases in a timely and rapid manner during the early stage of a pandemic is the safest measure for protecting individuals. It can minimize potential risks, such as containing outbreaks and reducing the peak pandemic size so that health care systems do not become overwhelmed [2,3,4], and buy time for research on viral vaccines. Normally, some nonpharmacological interventions are adopted to prevent disease diffusion during the early stage of the pandemic. For example, China implemented the principle of early detection, early reporting, early isolation, and early treatment to control COVID-19 and achieved some excellent results. Early detection is the key component for guiding the efficient prevention and control of an epidemic. The basic rules of nonpharmacological interventions primarily consist of three aspects: (1) removing virus sources, such as quarantining an infected person; (2) interrupting diffusion channels, such as maintaining social distance; and (3) protecting uninfected people, such as through home quarantine [5]. Identifying infected persons as quickly as possible is key to removing virus sources and further reducing the spread of the virus.

Screening people based on a detection rule is a general measure for identifying infected people. China’s nonpharmacological interventions for controlling COVID-19 during the early stage have shown that screening can efficiently support pandemic control efforts [2,6]. Some researchers have also illustrated the beneficial effects of screening measures, such as routine and early testing [7]. Screening is especially efficient for identifying asymptomatic persons who are infected. Research shows that 15% to 81% of cases of COVID-19 come from pre-symptomatic transmission [8]. Many cases cannot be detected by symptom-based testing due to the incubation period [9]. These infected persons will not go to the hospital or perform self-tests at home for diagnosis because they are unaware of their exposure to the disease.

Some screening strategies are adopted to identify infected cases early and have proven efficient, but they are considered conditional, slow-paced, and costly in specific scenarios. For example, paying more attention to high-risk people is a general screening strategy in the fight against COVID-19, such as health care workers [10], travelers [11], workers in airports or public areas, taxi drivers, and obstetric patients [12]. In China, the first infected person among 62.5% of COVID-19-diffusion emergencies from 2019 to April 2022 was discovered by proactive screening based on our rough estimation. The precondition of this type of proactive screening strategy is that the risks of this virus are clear; otherwise, some infected individuals may not be detected in a timely manner because of unknown risks. Thus, this strategy relies on knowledge of the virus. In addition, close contact tracing is also a screening strategy, and it has been shown to be efficient in many countries. However, close contact tracing requires the commitment of time and manpower. The accuracy and time span of traced histories for infected people are limited. Some scholars have indicated that close contact tracing is limited because only the contacts from a 2-day period before the onset of symptoms in the confirmed index case could be traced in most countries [13]. Some scholars believe that the contact tracing period should be extended to two weeks [8]. Moreover, generally, only the clustered family, travel or residence history, and contacts with infected persons are primary factors considered during close contact tracing [14]. Because of these features of close contact tracing, the efficiency of this approach is reduced when the infectivity of the virus is higher. In addition, mass screening has attracted the attention of many scholars. Random weekly mass testing was indicated to be effective in reducing the total number of infected people compared to no mass testing [15]. However, many papers have revealed that mass screening lacks sustainability, and they have stressed economy [5,16]. These measures are unlikely to be achievable in most countries around the world. The best strategy must be to detect the maximum number of asymptomatic cases early at minimal cost.

Although current screening strategies have worked, their weaknesses are apparent. Some authors expected 10–15% of cases to generate at least one unidentified secondary case that would need to be detected by other means [13]. Big data applications can efficiently close a gap in current screening strategies and can win time and manpower. Over the course of fighting COVID-19 in recent times, individuals with space–time intersections have been screened based on trajectory data. These persons refer to those who have intersections in space and time with infected persons during a specific time period. This strategy can efficiently support pandemic control work, and it also supports the significance and necessity of individual trajectory data. Many countries and regions have used people’s social interactions in movement tracking and potential infection screenings [17,18,19,20,21,22]. However, this strategy is another type of close contact tracing method, and it also depends on the appearance of symptoms in the confirmed index case. The difference is that the application of trajectory data improves the screening rate and efficiency of detecting asymptomatic persons.

To improve the screening efficiency, the features of network structures and dynamic transmissions can be used to develop efficient screening strategies. In this paper, we propose a strategy formulation framework (SFF) for developing efficient screening strategies based on trajectory networks. The essential idea behind the SFF is to identify the influential nodes in networks and utilize the influences of these nodes to find more infected individuals. SFF includes (1) rules for identifying influential persons according to network structures and dynamic diffusion features, (2) rules for setting screening priorities, and (3) rules for setting screening numbers. Based on the SFF, we propose two types of metrics to measure the influences of nodes and three types of screening modes to find more infected persons based on influential nodes. Then, we obtain six combinations based on the two metrics and three screening modes. That is, we propose six screening strategies according to SFF.

Moreover, to analyze evolutionary scenarios using different screening strategies, scenario simulation is adopted. Some scholars have developed and calibrated agent-based simulations to model COVID-19 outbreaks [23,24,25], and some studies have presented decision analysis tools to support policy makers by simulating different measure application scenarios [26]. To examine the efficiencies of the proposed strategies, we build a scenario model based on multi-agent modelling. According to the multi-agent modelling, the behavior state chart of persons needs to be designed, and we design three types of behavior state charts for simulating the normal life, information spreading, and virus diffusion of persons. According to behavior state charts, people have self-decisions, and they will move according to their decisions. A connection is generated if the distance between two persons is within the range of the infectious distance. That is, a virtual trajectory network is generated according to the contacts of people. This virtual trajectory network is used to calculate the influences of people.

To improve the reliability of our scenario model, most parameters are designed as interval values, and we use the Monte Carlo method to conduct our experiments to reduce the impact of randomness. We create two types of scenarios to examine our strategies, i.e., starting to screen when there is no confirmed case emergence yet and starting to screen after the first confirmed case has been discovered. The performances of the strategies in different scenarios are different although they all perform better than the scenario without any screening. The experimental results indicated that (1) when there is no confirmed case emerging yet, the persons who have more newly added contacts should have more attention (such as influential persons with the *NC_i_* metric; more details can be seen in Section 2.2), and screening persons according to their contact paths will have better performances. (2) When the first confirmed case has been discovered, the persons who have more contacts should be concerned with priorities (such as the influential persons with *M_i_* metric; more details can be seen in Section 2.2), and the sequential screening mode is more suitable to be used in this scenario. Further details are provided in the following sections.

## 2. Materials and Methods

### 2.1. Proposed Strategy Formulation Framework (SFF)

**Figure 1:**
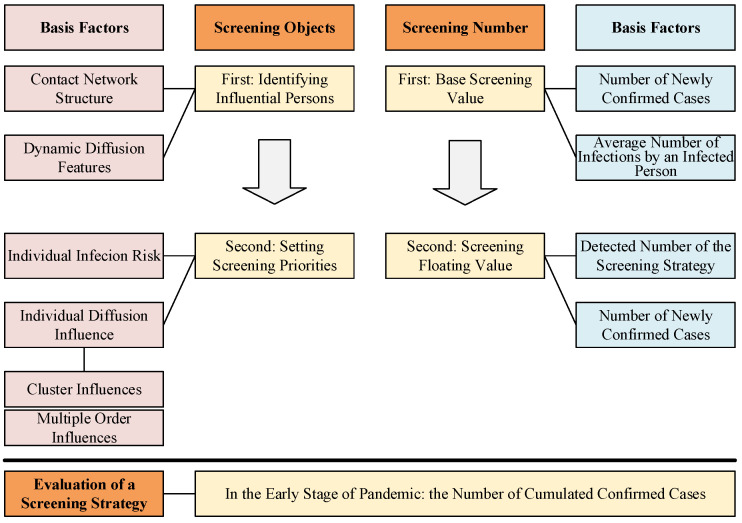
Illustration of the proposed strategy formulation framework (SFF). SFF is used to ensure the screening objects and screening number, and they all include two steps. The red column on the left illustrates the basis factors for identifying and selecting the screening objects. The blue column on the right illustrates the basis factors for setting the screening number. The bottom box illustrates the evaluation metric of screening strategy.

As shown in Figure 1, during a screening period, three questions need to be solved in a screening strategy: (1) Who will be screened based on which priorities? (2) How many people need to be screened? (3) How is the efficiency of a screening strategy evaluated?

#### 2.1.1. The First Question: Who Will Be Screened Based on Which Priorities?

The first step is to determine who is influential in virus diffusion. Individual people’s contacts spread the virus, while people’s activities generate new contacts [27,28]. People’s trajectory data present their activities and can be used to create a contact network [29,30]. In this network, nodes represent persons, and edges represent their contacts [31]. Because people’s activities are dynamic, this contact network is dynamic as well. Theoretically, a person with more contacts has a greater possibility of being infected or infecting others, and this person is influential in a crowd. Equivalently, there are influential nodes in a contact network [31]. Stronger influences increase the likelihood of virus diffusion. As a result, influential persons should be given more consideration when selecting screening objects. In addition, the structure and dynamic diffusion features of contact networks both affect people’s influences. Then, the second step is to define screening priorities according to the individual infection risk and diffusion influence. The former refers to the risk of a person becoming infected, and the latter refers to infected persons’ influence on others, such as the cluster influence (a local influence in a network) or the multiple order influence (a global influence in a network).

#### 2.1.2. The Second Question: How Many People Need to Be Screened?

The first step is to define the base screening value. Normally, the larger the screening number, the greater the chance of detecting infected cases, and the more helpful it will be in controlling the outbreak [32]. However, the limited resources available during the early stage of a pandemic restrict the number of people who can be screened. Sensibly, the screening number is associated with the number of newly confirmed people and the average number of infections by an infected person. More screenings are required to control the pandemic if the number of newly confirmed persons or the average number of infections by an infected person is higher. Moreover, the number of infected persons who have been screened during the previous period also has an impact on the screening number for the current period. Then, the screening number can be determined by (1) the number of newly confirmed persons, (2) the average number of infections by an infected person, and (3) the screened number of infected persons in the previous period.

The second step is to consider the floating value. The detected number of screening strategies and the number of newly confirmed cases are key reference indicators.

#### 2.1.3. The Third Question: How Is the Efficiency of a Screening Strategy Evaluated?

The scenario we are discussing in this paper is the pandemic scenario during the early stage, when people know very little about the virus. From the safest perspective, discovering all infected cases rapidly and in a timely manner is the best outcome. Of course, reducing the pressure of providing medical treatments or reducing the peak value of infected cases are also feasible approaches [33]; however, during the early stage, the evolution and cognition of the virus are not quite clear. Comparably, discovering all infected cases during the early stage is the least influential and safest approach. Therefore, in this paper, we use the cumulated confirmed number as an evaluation metric for a screening strategy.

In future work, more factors, such as the peak disease case number, the disease diffusion time, the cured number, and the costs, will be synthetically considered to present the control efficiency.

### 2.2. Proposed Screening Strategy

#### 2.2.1. Two Metrics for Identifying Influential People

According to the complex network structure, there are primarily three typical influences of nodes, i.e., (1) first-order influence, as measured by degree centrality, etc.; (2) hub influence, as measured by betweenness centrality, etc.; and (3) the multiple order influence, k-shell, etc. The influence of (1) is local for a node, and the influences of (2) and (3) are global for a node. Normally, local influences are more important than global influences for most virus diffusion in the short term. This is because the secondary infection rate and severity decrease with the increase of transmission generation. [34]. First-order and second-order infected persons usually receive more attention. Thus, the value of the k-shell can be assumed to be 2. Furthermore, considering that the infected possibility is affected by the contact frequency, the impact of the contact frequency should also be considered.

Then, this research proposes two metrics to evaluate the influences of people, i.e., $M_{i}$ and $NC_{i}$. 

(1) $M_{i}$ Metric

The $M_{i}$ metric, which is calculated using Equation (1), expresses the activity level of a person during a screening period. This activity level is measured by using the number and frequency of a person’s contacts and the number of his or her secondary contacts (i.e., the first-order and second-order influences of the person). In Equation (1), person *i* has contact with *No*. 1, *No*. 2, ……, and *No*. *n*. The contact number of person *i* is *n*; i.e., the degree of person *i* is *n*.

(1) $$M_{i}=\frac{\frac{c_{1}}{240}\times N_{1}+\frac{c_{2}}{240}\times N_{2}+\ldots \frac{c_{n}}{240}\times N_{n}}{Period}$$

The meanings of the parameters in Equation (1) are as follows:

(a) $c_{1}$ expresses the contact frequency between person *i* and *No*. 1;

(b) $N_{1}$ expresses the contact number of *No*. 1 within a screening period (i.e., the degree of *No*. 1 in the contact network);

(c) $\frac{c_{1}}{240}$ expresses the degree to which the contact time between person *i* and *No*. 1 is up to 4 h. $\frac{c_{1}}{240}$ will be smaller than 1 if the contact time is less than 4 h. (Some countries define a person as a contact who has spent four or more hours with a confirmed COVID-19 case [35]. Therefore, the time of 4 h is designed to measure the intimate degree of contact for the proposed strategies.);

(d) $\frac{c_{1}}{240}\times N_{1}$ indicates the activity level of person *i* after contacting *No*. 1. If person *i* is frequently in contact with *No*. 1, and *No*. 1 is also an active person who touches many people, then $\frac{c_{1}}{240}$ and $N_{1}$ are relatively larger. Then, person *i* has a higher risk because of the higher activity level;

(e) By that analogy, the overall activity level of person *i* can be determined by counting all their contact people. Period expresses the screening period, and $M_{i}$ expresses the average activity level of person *i*.

(2) $NC_{i}$ Metric

**Figure 2:**
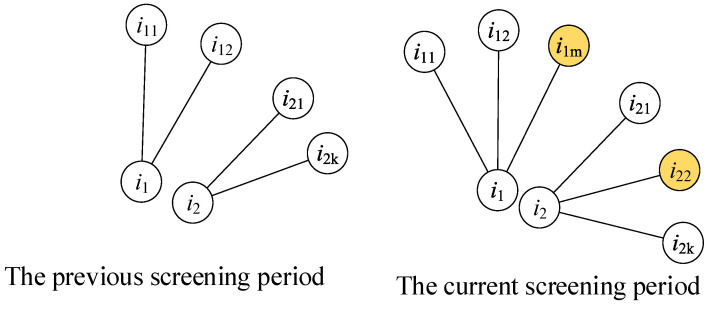
The measuring method for the $NC_{i}$ metric. The left sub-figure shows the contacts of person *i*_1_ and *i*_2_ in the previous screening period. The right sub-figure shows the contacts of person *i*_1_ and *i*_2_ in the current screening period. The person *i*_1*m*_ and *i*_22_ are two newly added contacts of person *i*_1_ and *i*_2_.

The $NC_{i}$ metric is calculated according to the contact numbers of a person before and during the current screening periods, as shown in Figure 2, and $NC_{i}$ expresses the new contact numbers of a person during the current screening period. The contact persons $i_{1m}$ and $i_{22}$ are the new contact people of the two individuals $i_{1}$ and $i_{2}$, respectively, and both $NC_{i}$ values of the two people are 1.

$NC_{i}$ presents the dynamic features of the contact network. A person has a larger $NC_{i}$ if they have contact with more new people, which means that this person has a dynamic hub influence. Similar to the attribute of betweenness centrality, this person has a larger possibility of contacting uninfected people.

#### 2.2.2. Setting Screening Priorities for Influential People

##### Setting Screening Priorities Based on Individual Infection Risk

In the SSF, people’s influences (i.e., metric values) are used to present their infection risks. Normally, individuals who have higher infection risks should be screened and detected with priority. The screening mode using this logic is named the sequential screening mode. This mode only considers the activity levels and new contact numbers of the subjects and ignores the characteristics of the contact network structure.

##### Setting Screening Priorities Based on the Individual Diffusion Influence

The diffusion influences of individuals on others should also be considered for pandemic control. There are two types of individual diffusion influences, i.e., the local diffusion influence (cluster influence) and the global diffusion influence (multiple order influence).

(1) Cluster influence

Cluster influence is one type of typical local influence in a complex network, and it is significant in virus diffusion. The cluster coefficient is used to evaluate the aggregation of nodes [36]. The essential idea behind the cluster coefficient is to calculate the number of triangles consisting of nodes and edges. Family members are in contact with each other, and a family is usually a cluster of family members. Moreover, the degree centrality also represents the local influences of nodes. People with more connections have larger local influences. Thus, having more family members means more family connections, which further implies having a higher cluster influence and a higher infection risk. This paper exploits family relationships to present the cluster influence of a person, and this screening mode is named the family cluster screening mode.

Families with more members were screened first. For example, a family with five members has priority for screening compared to a family with two members. In addition, to improve the cooperation of the family cluster screening mode with other screening strategies efficiently, only one person would be screened in a family, and the screened person would be selected according to the ranking of family members based on individual infection risks. If there is a situation in which multiple individuals have the same individual infection risk, this paper assumes that the ranking of these individuals would be determined by experimental randomness.

(2) Multiple order influence

**Figure 3:**
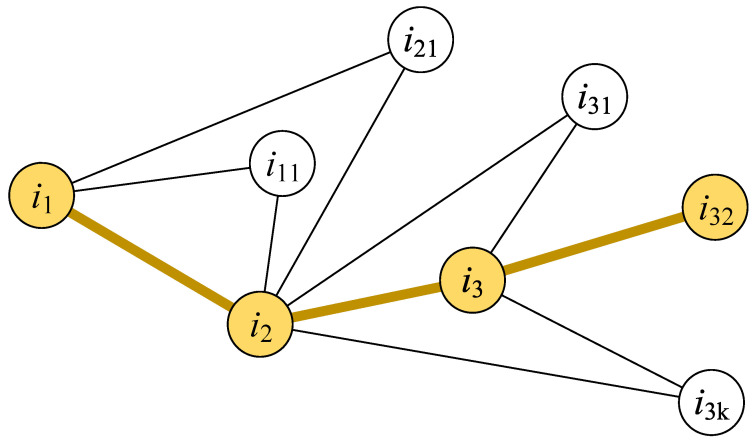
Illustration of the long-line screening mode. The yellow nodes are selected persons who are screened, and the yellow lines between nodes expresses the contact path between persons.

The nodes’ global influence can be presented by their effects on multiple order nodes. Similarly, the global influence of an infected person is higher if the person can infect more individuals through multiple connections [37]. Based on this idea, we propose the long-line screening mode. As shown in Figure 3, the essential idea behind the long-line screening mode is to select the connected person who has the largest individual infection risk at each connection level. First, we should determine which contact line should have priority selection. All people are sorted according to individual infection risks, and the person with a larger individual infection risk will be selected as the source person (*i*_1_) of the screening. Then, the next person ($i_{2}$) will be selected from the contact persons of the source person (*i*_1_). The contact person who has the most noncommon neighbors with the source person (*i*_1_) will be selected. The contact person who has the most non-common neighbors could infect more people who are not in contact with the source person (*i*_1_); this type of contact person has a higher hub influence on the source person (*i*_1_) and the neighbors of the contact person ($i_{2}$). The next selected person ($i_{3}$) will be selected in the same way until there is no next contact person to be selected in this contact line. The priority is to detect the above-selected people in multiple connections.

We designed a rule to coordinate the application of multiple screening strategies efficiently. That is, if people are selected using the above three modes, their families will not be selected since the families will be detected by close contact tracing once the selected individuals become confirmed cases.

In summary, according to the aforementioned metrics and modes, six combinations are generated:

(a) Sequential screening by $M_{i}$ value of people in a screening period (Seq-M for short);

(b) Long-line screening by $M_{i}$ value of people in a screening period (LL-M for short);

(c) Family cluster screening by $M_{i}$ value of people in a screening period (Fam-M for short);

(d) Sequential screening by $NC_{i}$ value of people in a screening period (Seq-NC for short);

(e) Long-line screening by $NC_{i}$ value of people in a screening period (LL-NC for short);

(f) Family cluster screening by $NC_{i}$ value of people in a screening period (Fam-NC for short).

#### 2.2.3. Screening Number in a Screening Period

According to the SSF, we designed a base screening value and a floating value for the screening number in a screening period. In addition, we assume that the upper limit of the screening number is 20% of the population.

##### The Base Screening Value in a Screening Period

The base screening value $N_{detection}$ depends on the number of newly confirmed cases and *R* within a screening period. As shown in Equation (2), $N_{detection}$ equals the product of the number of newly confirmed cases and *R* in the current period. As presented in Equation (3), *R* is the average number of infections by a confirmed person, and *R* is dynamic. During the initial screening period, *R* and the number of newly confirmed cases cannot be known immediately; thus, we assume that $N_{detection}$ equals the number of newly confirmed cases if *R* is unknown, or *R* is smaller than 1 (see Equation (2)), and $N_{detection}$ equals 5% of the people size if the number of newly confirmed cases is zero (see Equation (2)). The settings of “5%” and “20%” are assumptions, and the values of these two parameters can be designed to fit various real-world scenarios. In our experiments, these parameters’ settings cannot affect the experimental results and conclusions because they are controlled variables when conducting the compared experiments.

(2) $$N_{detection}=\left\{\begin{array}{c} N_{newCases}\;\;\;\;\;\;\;\;\;\;\;\;\;\;R=infinity\;\;\left|\;\right|\;R<1\;\;\;\;\;①\;\;\\ N\times 5\%\;\;\;\;\;\;\;\;\;\;\;\;\;\;\;\;N_{newCases}=0\;\;\;\;\;\;\;\;\;\;\;\;\;\;\;\;\;\;\;\;\;\;\;②\;\;\\ N_{newCases}\times R\;\;\;\;\;\;\;\;R\geq 1,\;N_{newCases}>0\;\;\;\;\;\;\;③\;\; \end{array}\right.$$

where *N* denotes the number of people, and $N_{newCases}$ expresses the number of newly confirmed cases in the current screening period.

(3) $$R=\frac{\sum_{N_{infected}}^{i}N_{i}^{infectious}}{N_{infected}}$$

where $N_{infected}$ expresses the current total number of infected people, $N_{i}^{infectious}$ denotes the number of infections by infected person *i*, and $\overset{i}{\underset{N_{infected}}{\sum }}N_{i}^{infectious}$ expresses the total number of infections by all infected people.

##### The Screening Floating Value in a Screening Period

The screening floating value is used to adjust the screening number in a screening period dynamically. If the detected confirmed number from the previous screening period by screening strategies is smaller than the number of newly confirmed cases, then the screening number in the current screening period will increase by 1% of the population size within the upper limit. If the confirmed number detected by screening strategies in the previous period exceeds the number of newly confirmed cases in the current period, the screening number will remain $N_{detection}$. We expect that the settings of the screening floating value will efficiently screen more infected persons.

### 2.3. Proposed Model

To simulate the pandemic process and deduce the efficiencies of screening strategies, this paper builds and proposes a scenario model based on multi-agent modeling. This model incorporates people’s activity features and typical behaviors, in which some constructed settings are as follows: people have their family members, family addresses, and workplaces. In addition, there are some gathering areas, such as malls, markets, and clubs. Using their various behaviors, activity states, and interactions, we simulate people’s heterogeneity and further obtain their trajectory data from the simulation process.

Three modules are designed to describe the activity features and behaviors of people: (1) normal life, (2) disease diffusion, and (3) information spreading. In each module, we design a state chart to simulate the typical behaviors of one person, and different people may be located in different states at the same time during a simulation, and people’s behaviors are affected by others. That is, this is a nonlinear system including various interactions of people. According to this characteristic, crowd features emerge.

#### 2.3.1. The Activity Features and Behaviors of People in Normal Life

**Figure 4:**
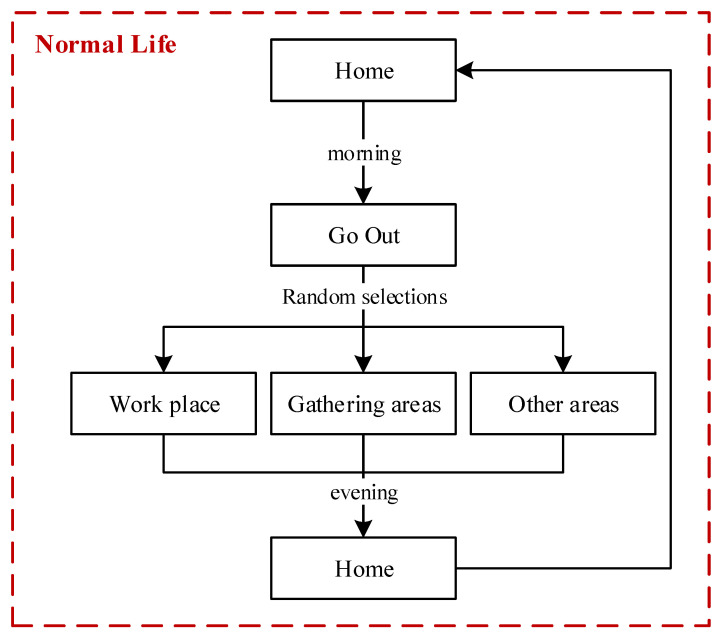
Flow chart of a person living a normal life.

In our model, people who do not have symptoms of infection would continue their normal life. In the normal life of one person, three typical characteristics are designed: (a) a person may leave home in the morning or choose to stay home; (b) a person will go home in the evening if this person is outside; and (c) the daily travel range of a person includes workplaces, gathering areas, and residential zones. The flowchart of a person living their normal life is illustrated in Figure 4.

#### 2.3.2. Activity Features and Behaviors of People during Disease Diffusion

In our model, we designed a mechanism for people’s decisions during the pandemic process, which involves the fields of working, moving, infecting, isolating, and curing. Based on this mechanism, two people may generate infections if they choose to move and are located in the same space region at the same time, which means that this infection is created by their own decisions. In our model, the microscopic mechanism is designed, and the macroscopic features emerge through people’s interactions.

**Figure 5:**
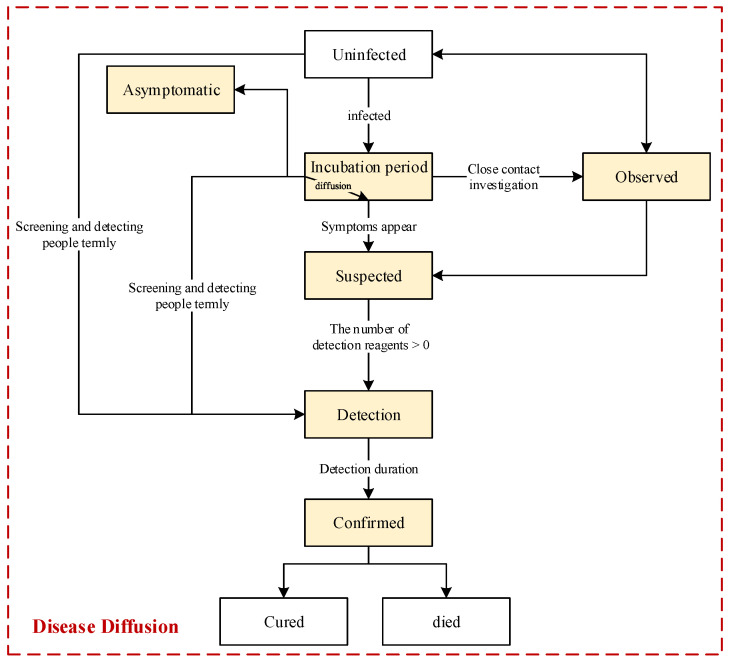
Flow chart of disease diffusion. The states of people will change when the transition conditions are met. The transition named “diffusion” indicates that the virus would be diffused by infected persons when they are active in the state of “incubation period”. Moreover, (1) to use as few resources as possible, only contacts of confirmed cases would be detected by close contact tracing in this mode; (2) we assume that people will have permanent immunity after being infected with the virus in this model, i.e., considering only the situation of one round of virus diffusion.

As shown in Figure 5, we designed the flow chart of disease diffusion. In the beginning, all the people are uninfected, and the first infected person emerges when exposed to the virus from areas outside of this crowd or when in contact with an infectious source in the environment. The infected person must experience an incubation period before the disease symptoms appear. During this period, the infected person has the potential to diffuse the virus to contact persons. When an infected person is viewed as a suspected case after developing disease symptoms or as a close contact person through epidemiological investigation, this person would be and needs to be detected. Hypothetically, infected people could be discovered and listed as confirmed cases once they are detected, and they would start to be treated and would be cured or would die after a treatment period.

Additionally, there are some asymptomatic individuals in the crowd because they do not exhibit any disease symptoms all the time. Some of these asymptomatic people can be discovered through close contact tracing or screening strategies. Figure 5 shows that the people who have been selected will be directly detected by using screening strategies; i.e., the infected persons among them, regardless of whether they are asymptomatic or in the incubation period, will be discovered. Moreover, these people are discovered in advance, and accordingly, their curability could be higher. Therefore, people’s curability is associated with the duration between the time they are infected and confirmed.

In addition to the above features, we also assume that the observed, suspected, and confirmed individuals would be isolated in fixed places until they have no proven infection. Hypothetically, there is no more infection in the fixed isolations. Furthermore, considering that the incubation period of an individual is related to the person’s immunity, we assume that the incubation period will be longer if the person’s immunity is stronger, and an infected person can be asymptomatic if his or her immunity is strong enough.

Moreover, in this model, we indicate that an infected person could infect a contact person if the distance between the two is within a range of possible transmission. Clearly, as everyone knows, there is no infection if two people have an adequately safe distance. According to this design, prevention measures for maintaining social distancing can also be designed during the pandemic process.

#### 2.3.3. Activity Features and Behaviors of People during Information Spreading

The implementation of some prevention measures, such as maintaining social distance, can be effective only if people consciously execute them. In our model, we exploit information spreading among people to improve their awareness. People who believe warnings would have high consciousness about maintaining a safe social distance.

**Figure 6:**
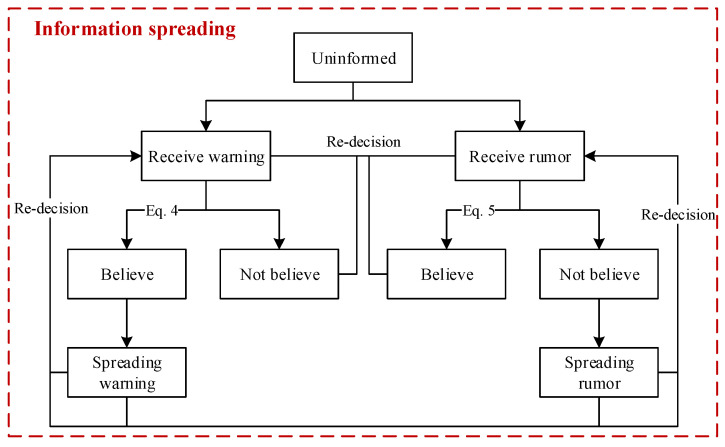
Flow chart of information spreading. The states of people will change when the transition conditions are met. Some transitions are described as equations.

As illustrated in Figure 6, a flow chart of information spreading among people was designed. In the beginning, all the people are uninformed. Then, several persons in a crowd receive a warning when pandemic control starts, and this warning delivers the information that people should have the consciousness to prevent disease diffusion. We assign a value to the warning, which ranges from 0 to 1 (i.e., $V_{infor}$). A warning with a higher value is more likely to be trusted. Moreover, a person can receive information spread by different persons. Thus, the values of information received by a person can be cumulated (i.e., $C_{infor}$). Whether people believe the warning depends on them. Cognition about the disease and the current disease diffusion situation, such as the currently confirmed number, jointly affect people’s decisions. As presented in Equation (4), people make decisions through the comparison of $T_{warning}$ and $C_{infor}$.

(4) $$T_{warning}=\left(1-P_{cognition}\right)\times (1-\frac{N_{currentlyconfirmed}}{N})$$

where $P_{cognition}$ expresses the cognition of a person about the disease; $P_{cognition}$ is larger if a person prefers to prevent and control the disease diffusion, and people differ in their values of$\;P_{cognition}$. $N_{currentlyconfirmed}$ denotes the currently confirmed number, and *N* is the number of people. A person has a higher possibility of believing this warning if he has a higher cognition of the disease, or the currently confirmed number is larger, i.e., $|C_{infor}|\;\geq T_{warning}\&\&\;C_{infor\;}>0\;$($C_{infor\;}>0$ denotes that this information is a warning). Hypothetically, in this model, individuals who have been warned will randomly spread warnings to contact persons, and subsequently, the contact persons will receive warnings and decide whether to believe the warnings.

In addition, there are rumor disseminators during warning spreading, and people can receive warnings and rumors simultaneously. Similarly, a person who has received a rumor decides whether to believe it, and the decision depends on the person’s cognition and the current disease diffusion situation. As indicated in Equation (5), people will believe the rumor if $|C_{infor}|\geq T_{rumor}\;\&\&\;C_{infor\;}<0$ ($C_{infor\;}<0$ denotes that the information is a rumor); otherwise, people will not. People who believe rumors will disseminate them to others.

(5) $$T_{rumour}=P_{cognition}\times \frac{N_{currentlyconfirmed}}{N}$$

where $T_{rumour}$ denotes the threshold for making decisions.

$C_{infor}$ of a person will add value if this person receives a warning with the value. By contrast, a value will be subtracted from $C_{infor}$ if this person receives a rumor. The symbols “plus” and “minus” indicate that the information someone has received is a warning or a rumor, respectively. A person may not believe any information if the warnings and rumors that he has received are almost even. During this process of intermingling warnings and rumors, people’s degree of belief in the information they receive is dynamic. People who have believed the warning may also receive rumors, and they can also be affected to believe the rumor. In the same way, a person who has believed the rumor can also be affected to believe a warning after he receives warnings. Of course, with the influence of rumors and warning dissemination, people may believe neither rumors nor warnings.

Moreover, the value of information may change during the process of information spreading because of various interpretations of information by different people. The value of information may increase when people exaggerate the information’s meaning, and the value of information may decrease when people do not completely express the information’s meaning.

Furthermore, in this paper, people spread warnings or rumors through the contact network, and the online spreading network has not been considered in the current scenario model. In the future, we will continue to expand our model to a double-level network, which includes a contact network to support disease diffusion and an online social network to support information spreading.

### 2.4. Experimental Designations

We want to build a scenario of a pandemic caused by a virus that was not previously known. In this scenario, the pandemic of this virus happens for the first time. All the parameters and their values in the model are listed in Table 1. According to the self-similarity of the complex system, a population size of 2890–3100 (1000 families) is designed in our model and experiments to improve the computational efficiency as much as possible. The compared experiments will present the optimal screening strategy for disease prevention and control. In this model, an infected person is assumed to infect their contact people according to a rate (a possibility value conforms to the uniform distribution of 0–1) per minute. We use the Poisson or normal possibility distributions to set the parameters of our model to perform various decisions and behaviors of individuals. In real life, most people’s decisions conform to bell-shaped possibility distributions [38,39]; i.e., most people make nearly the same decisions, and only a few people display extremely different decisions or behaviors.

Most parameters are designed as ranges to present the heterogeneity of individual people. We randomly assign individuals the number of family members, each of which is one to five persons. In our experiments, hypothetically, the detection reagents are adequate. Within an infectious distance, two people may generate an infection connection and create an infection.

The parameter, “Initial infected persons in a day”, indicates that newly infectious sources (such as initially infected persons) always appear in the crowd before prevention. We assume that the virus source in the environment will be discovered and controlled once the related prevention and control measures start to be executed. That is, in the case of starting to perform preventions, initially infected persons no longer appear.

The parameter, “New spreaders of warning/rumor in a day”, expresses that new warnings or rumor spreaders always appear in the crowd after warnings and rumors start to be diffused in the crowd. These new spreaders may receive information from online networks, radios, TV, information delivery platforms, or other channels outside of the crowd.

Moreover, the mortality of an infected person is associated with the time when this person is confirmed. Thus, the mortality of an infected person is lower if their infection is discovered and confirmed earlier. In addition, the incubation duration of an infected person is related to their immunity. Specifically, an infected person who has higher immunity will have a longer incubation duration if the infection is not discovered in time. If the immunity of an infected person exceeds 0.9, then this infected person is designated as an asymptomatic person (this is an assumption).

In addition, our model simulates some random decision behaviors (such as whether to go out, where to go today, and whether or not an infection is created between two contact persons), and we use random functions, such as normal possibility distributions of 0–1, to implement these behaviors.

In addition, the parameter, “Close contact tracing rate”, expresses the time interval of people’s memory in close contact tracing. During close contact tracing, an investigated person needs to remember and describe his or her contact history. We designed the time interval of his or her memories to be 2 h. Therefore, the person needs to describe his or her contact history with others every 2 h in the past. Then, how many days need to be remembered? In this model, we assume that a person can remember the contact history of all the simulation times. For example, a person is investigated to determine whether they have had contact with an infected person, and the simulation time is 10 h. Then, that individual can remember and describe their contact history at the 8th hour, 6th hour, 4th hour, and 2nd hour. They cannot describe their contact memories from other times. In this case, the efficiency of close contact tracing depends on the close contact tracing rate. Of course, close contact tracing would be more accurate if the rate were designed to be 1 min. However, 1 min is ideal and does not conform to real situations. On the basis of this designation, some infected cases could not be picked up by close contact tracing.

As shown in Table 2, 16 experiments are designed, and the experimental scenes include the following:

(1) Disease diffusion with no application of any screening strategy (i.e., no action);

(2) Disease diffusion in which only the preventive measure of maintaining social distancing is used;

(3) Disease diffusion in which the random screening strategy is applied based on social distancing;

(4) Disease diffusion in which different proposed screening strategies are applied based on maintaining social distance.

The simulation time is designated as 91 days. According to our preliminary experiments, disease diffusion can evolve throughout the rising, plateau, declining, and post-pandemic periods over 91 days. To reduce the impact of randomness, the Monte Carlo method is used in the experiments. In a Monte Carlo experiment, the parameter settings of all simulations are the same, and the only difference is the random number. We can generate various evolutionary scenes with the same initial settings by using different random numbers. Each simulation will be repeatedly conducted 20 times with 20 random numbers in a Monte Carlo experiment. In addition, according to the SSF, the cumulated confirmed number is used to represent the efficiency of the applied disease control measures.

To observe efficiencies using different screening strategies in different scenarios, we apply these proposed screening strategies to two scenarios: (1) the first scenario is applied after the first confirmed case appears (i.e., using strategies only for an emergency), and (2) the second scenario is applied before the first confirmed case appears (i.e., using strategies during a normal situation and an emergency).

## 3. Results

In this section, we present the experimental results of the two experimental scenarios. According to the SSF, the cumulated confirmed case number is used to evaluate the screening strategy in this paper. In a Monte Carlo experiment, 20 simulations will generate 20 cumulative confirmed cases. The relative frequency distribution of the 20 confirmed numbers is used to illustrate the efficiencies of the screening strategies.

### 3.1. Experiment Results of the 1st Scenario

**Figure 7:**
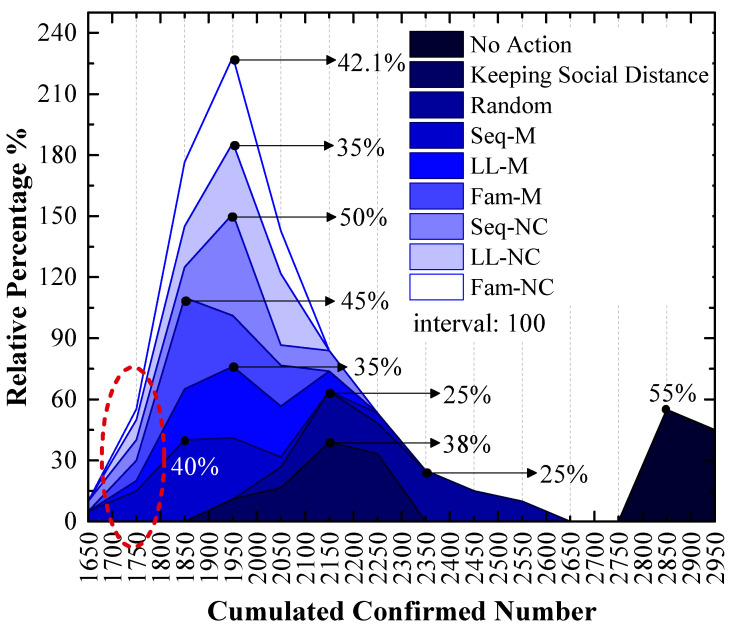
The relative frequency of cumulated confirmed cases using various strategies in the 1st scenario. The x-values of the peak values express the cumulative confirmed number with the highest probability.

As shown in Figure 7, which is a stacked area figure, the applied screening strategy is more efficient when the x-value of the peak point is smaller, and the applied screening strategy is more efficient with a larger y-value for the peak point when the x-values are the same.

Figure 7 shows the following:

The order of x-values for the peak points obtained by using different measures from small to large is Seq-M (1850) = Fam-M (1850) < LL-M (1950) = Seq-NC (1950) = LL-NC (1950) = Fam-NC (1950) < Keeping Social Distance (2150) < Random (2150, 2350) < No Action (2850). This order indicates that compared to the result without any measure, using the proposed strategies can reduce the number of confirmed cases by at least 31.58%, and it can reduce confirmed cases by at most 35.09% with the largest probability. Maintaining social distance can reduce confirmed cases by 24.56% with the largest probability. Additionally, the random screening strategy performs worse than the measure of maintaining social distance.

Seq-M and Fam-M have the same x-value for the peak points, and the stacked y-value of the peak point obtained by using Fam-M (45%) exceeds that obtained by using Seq-M (40%). The order of y-values for peak points obtained by using other proposed strategies from large to small is Seq-NC (50%) > Fam-NC (42.1%) > LL-M (35%) = LL-NC (35%).

**Figure 8:**
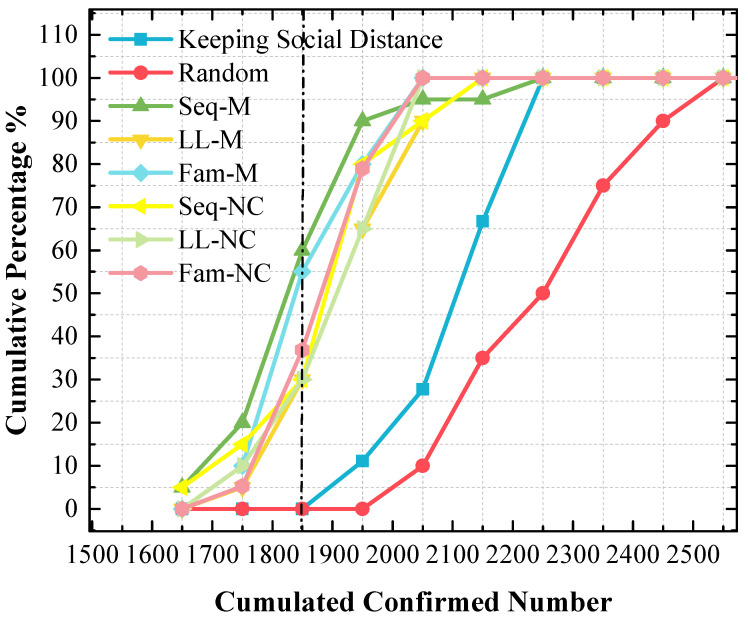
The cumulative frequency of cumulated confirmed cases using various strategies in the 1st scenario. The x-value of the dash line is 1850.

Although the stacked y-value of the peak point obtained by using Fam-M exceeds that obtained by using Seq-M, Seq-M is the most efficient strategy. As shown in the red circle of Figure 7, the stacked area before the peak point obtained by using Seq-M is larger than that obtained by using Fam-M. More simulation results are distributed to the left of the peak point obtained by using Seq-M. As shown in Figure 8, when Seq-M is used, the cumulative percentage with the 1850 x-value is larger than that when Fam-M is used.

**Figure 9:**
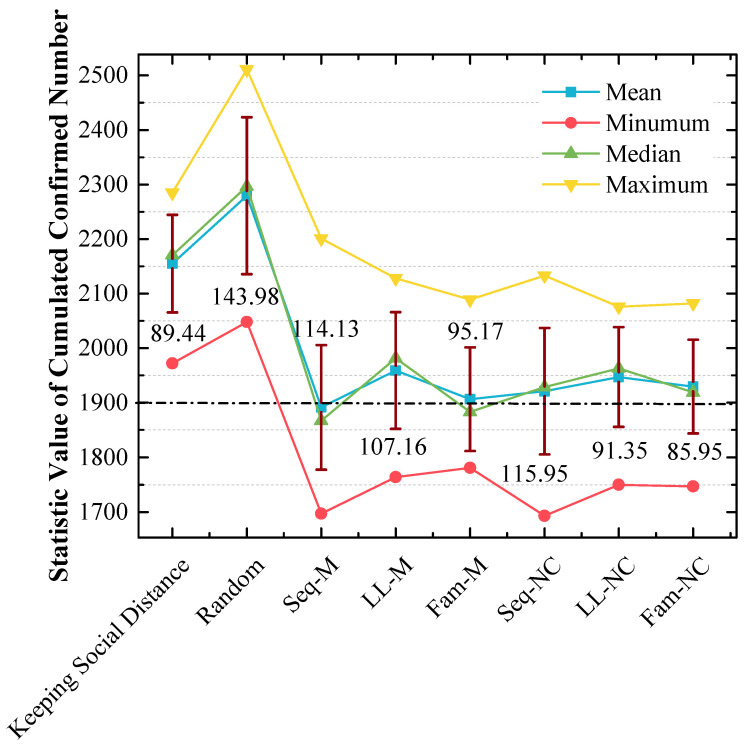
Statistical analysis of cumulated confirmed numbers using different strategies in the 1st scenario. The red lines express the standard variances of cumulative confirmed numbers.

In addition, 20 cumulative confirmed numbers for each strategy are statistically analyzed in Figure 9, which shows the following:

(a) The means and medians are approximately 1900 when Seq-M and Fam-M are used, and the mean and median are slightly smaller when Seq-M is used. Moreover, the means and medians are larger than 1900 when other strategies are used. In addition, when the long-line screening mode is used (i.e., LL-M and LL-NC are used), the means and medians are approximately 1950, which are the largest;

(b) The order of standard variances obtained by applying different strategies from large to small is Seq-NC (115.95) > Seq-M (114.13) > LL-M (107.16) > Fam-M (95.17) > LL-NC (91.35) > Fam-NC (85.95).

### 3.2. Experiment Results of the 2nd Scenario

**Figure 10:**
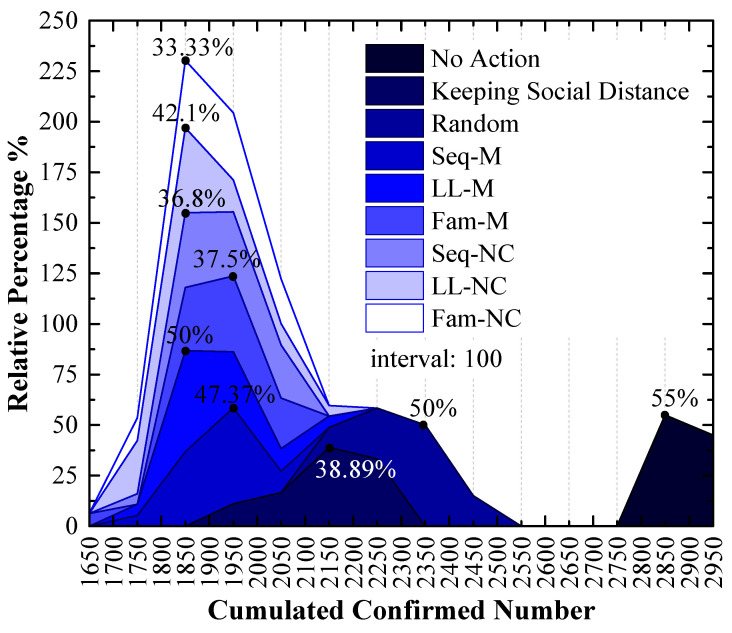
Relative frequency of cumulated confirmed numbers using different strategies in the 2nd scenario. The x-values of the peak values express the cumulative confirmed number with the highest probability.

**Figure 11:**
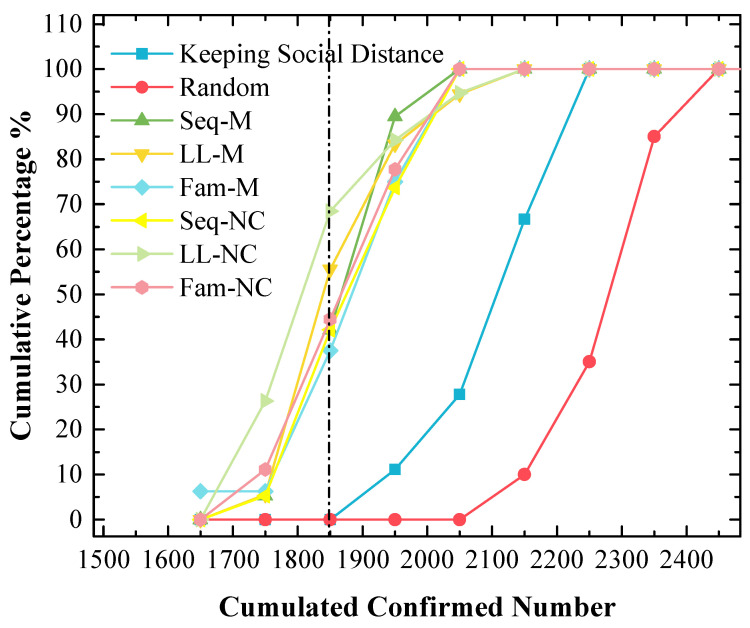
The cumulative frequency of cumulated confirmed numbers under different strategies in the 2nd scenario. The x-value of the dash line is 1850.

**Figure 12:**
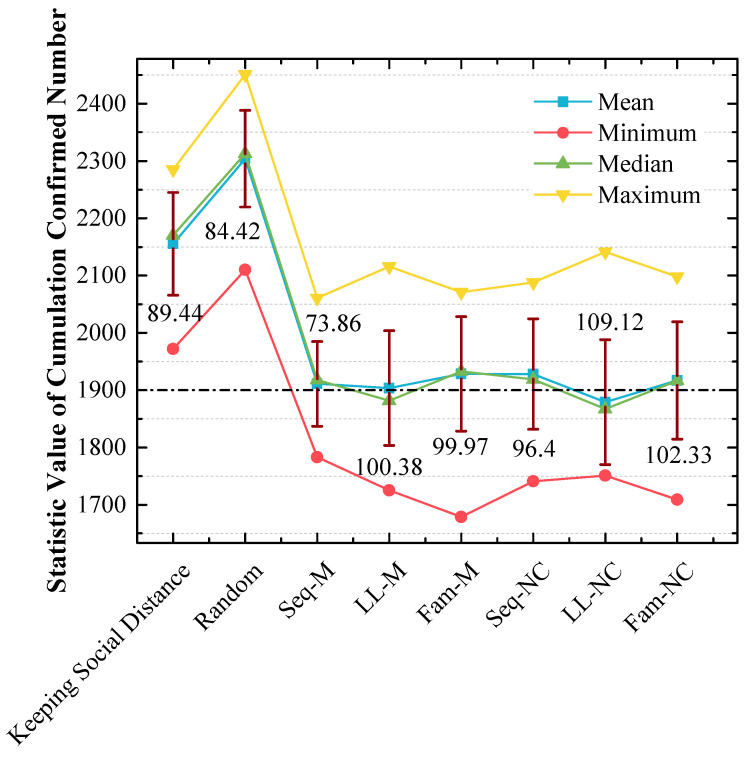
Statistical analysis of cumulated confirmed numbers using different strategies in the 2nd scenario. The red lines express the standard variances of cumulated confirmed numbers.

In the 2nd scenario, the experimental results are illustrated in Figure 10, Figure 11 and Figure 12. As shown in Figure 10, the peak points of four screening strategies are distributed around an x-value of 1850, and only two strategy peak points are distributed around an x-value of 1850 in Figure 7.

Figure 10, Figure 11 and Figure 12 show the following:

(a) In Figure 10, the order of x-values for peak points by using different strategies from small to large is LL-M (1850) = Seq-NC (1850) = LL-NC (1850) = Fam-NC (1850) < Seq-M (1950) = Fam-M (1950) < Keeping Social Distance (2150) < Random (2350) < No Action (2850). The strategy order of y-values for peak points with the 1850 x-value from large to small is LL-M (50%) > LL-NC (42.1%) > Seq-NC (36.8%) > Fam-NC (33.33%);

(b) In Figure 11, the y-value is almost 70% with the 1850 x-value when LL-NC is used, and the y-value is almost 60% with the 1850 x-value when LL-M is used;

(c) Unlike the results in Figure 9, in Figure 12, the strategy order of means and medians from small to large is LL-NC < LL-M < Seq-M < Fam-NC < Seq-NC < Fam-M. The performance of LL-NC and LL-M is better. The strategy order of standard variances from large to small is LL-NC (109.12) > Fam-NC (102.33) > LL-M (100.38) > Fam-M (99.97) > Seq-NC (96.4) > Seq-M (73.86).

## 4. Discussion

In this section, we discuss the performances of different screening strategies for pandemic prevention and control according to the experimental results.

### 4.1. Seq-M Is the Most Efficient Strategy in the 1st Scenario

Figure 7 and Figure 10 show that our proposed screening strategies are efficient during the pandemic.

In the 1st scenario, compared to the situation in which no measure is used, disease diffusion can be controlled very well by using any measure, and the results show that disease diffusion can be mitigated further by using the proposed screening strategies on the basis of maintaining social distance, especially Seq-M and Fam-M, which are more efficient and can reduce the number of confirmed cases. The result of Figure 8 indicates that Seq-M is the most efficient because more simulation results are distributed to the left of the peak point obtained by using Seq-M, thus indicating that the control efficiency is more likely to be better by using Seq-M.

Figure 9 shows the results of 20 cumulative confirmed numbers for each strategy. The results also confirm that Seq-M is the most efficient because Seq-M has the smallest mean and median.

### 4.2. LL-NC Is the Most Efficient Strategy in the 2nd Scenario

First, from the results of Figure 7 and Figure 10, it can be concluded that disease diffusion can be further efficiently controlled when these screening strategies are used before the first confirmed case appears.

In the 2nd scenario, Figure 10 indicates that LL-NC can further reduce the cumulated confirmed number (the stacked y-value of the peak point is 42.1%), and Figure 11 demonstrates that more results are distributed to the left area of the peak point of LL-NC (the cumulative percentage is 70% with the 1850 x-value). Therefore, LL-NC is the most efficient strategy in the 2nd scenario. The result of Figure 12 also supports this conclusion; i.e., LL-NC has the smallest mean and median of cumulated confirmed numbers.

### 4.3. Long-Line Screening Mode Does Not Perform Well in the 1st Scenario and Performs Well in the 2nd Scenario

In the 1st scenario, as shown in Figure 9, compared to other screening strategies, the means and medians obtained by using LL-M and LL-NC are larger. In Figure 8, major simulation results are distributed to the right of the peak point obtained by using LL-M and LL-NC (i.e., the cumulative percentages are approximately 30% with the 1850 x-value.) Therefore, it can be concluded that the long-line screening mode does not perform well in the 1st scenario.

**Figure 13:**
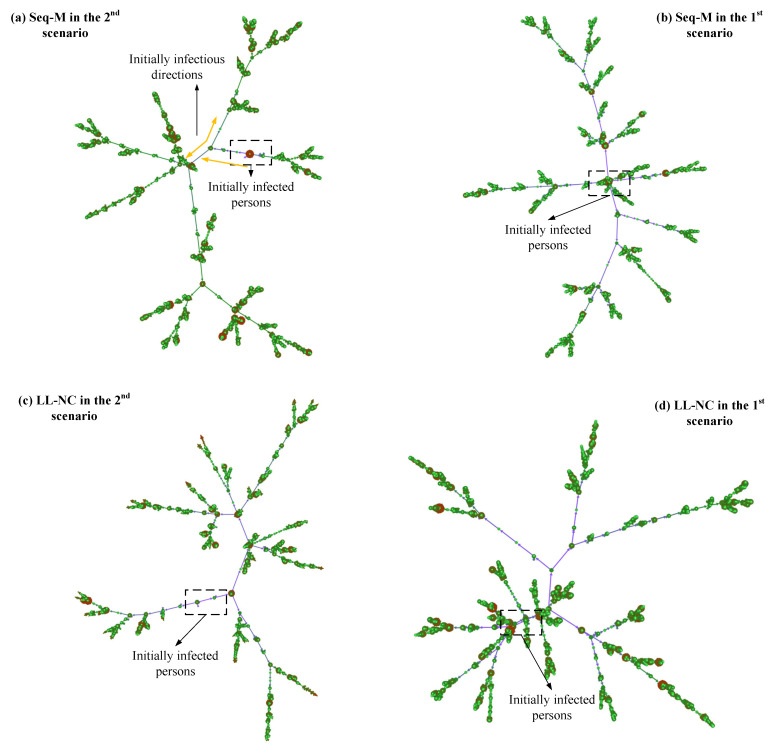
Diffused networks using Seq-M and LL-NC in the two scenarios. These are directed networks. The larger the degrees of nodes, the larger the diameter of circles. The more nodes are clustered, the larger the density of the connections between nodes.

Why do LL-M and LL-NC perform poorly? In the 1st scenario, screening strategies are applied after the first case appears, thus indicating that the virus has been diffused for some time, and some diffused lines have been formed. In this situation, the individuals screened using the long-line screening mode may have already had their first infections, and the effects of infecting others have already occurred. In this situation, avoiding more infections from them is the primary concern [40]. However, the contact lines screened by using the long-line screening mode are limited, and it is difficult to screen all diffused lines that have formed. As shown in Figure 13d, more bifurcated diffused lines are formed from the original diffused lines. Some infected people may not be screened, which leads to poor efficiency in extracting more infected cases in advance.

In contrast, in the 2nd scenario, LL-NC and LL-M have great performance. When screening strategies are applied before the first confirmed case appears, the diffused lines have not yet formed, their lengths lines are short, and their number is small. In this situation, determining the infected persons by following the contact lines is efficient and precise, and more infections by the contact individuals can be avoided in advance [41]. Furthermore, it is possible to discover most infected people using this approach because of the small number and short lengths of diffused lines. This is especially true when using the $NC_{i}$ metric. The people who will diffuse the virus to other small clusters can be identified in advance by the $NC_{i}$ metric, and these people may speed up the growth of the diffused lines. Identifying these people early can efficiently interrupt diffusion and reduce diffused lines. As shown in Figure 13c, compared to the result in Figure 13d, there are fewer or shorter bifurcated diffused lines.

### 4.4. Seq-M and Fam-M Perform Well in the 1st Scenario and Do Not Perform Well in the 2nd Scenario

In the 1st scenario, from Figure 7 and Figure 10, it was observed that Seq-M and Fam-M are the most efficient, and in contrast, Seq-M and Fam-M perform the worst in the 2nd scenario.

The only difference between the two results is the different applied scenarios. According to our above discussions, some diffused lines have grown for a while in the 1st scenario, which means that more people have been infected. Then, the infected individuals in the diffused lines would extend their diffusion in the local range. The increase in infectious edges in the diffused network depends on having more contacts with infected peoples. The individuals screened by Seq-M and Fam-M have more local contact with others, and they have more possibilities of contacting infected people or infecting others. Screening people according to the larger $M_{i}$ metrics or larger families provides higher accuracy. As shown in Figure 13b, compared to the results in Figure 13a–d, there are fewer red nodes in diffused lines (the red node has larger infections with others), which indicates that the infected individuals did not extend the virus over a wider range, and the people with larger numbers of contacts have been screened before they infect others when applying Seq-M in the 1st scenario.

In the 2nd scenario, initially, diffused lines have not formed. The growth of diffused lines does not depend on the local contacts but on multiple contacts. However, the $M_{i}$ metric only expresses a local influence. Therefore, screening people by $M_{i}$ metrics could have lower accuracy. As shown in Figure 13a, the initially formed diffused lines primarily rely on the nodes with smaller-degree nodes and not larger-degree nodes. (The smaller-degree denotes fewer infections with others, and the light-colored nodes have a smaller degree.)

Moreover, when using $M_{i}$ metrics, if a screened person is infected, initially, the infected person may not infect others yet, but many contact persons of the screened person need to be traced by close contact tracing. They are isolated in a fixed place. Although there is no more infection in isolation, they could be infected when they are moving to isolation. (According to our design, the traced individuals are required to move into isolation.) Thus, as shown in Figure 13a, compared to the result in Figure 13b, there are more red nodes in the diffused lines. We believe that this is the reason why Seq-M performs more poorly in the 2nd scenario than in the 1st scenario, and the performance of Fam-M conforms to the same logic.

### 4.5. The Stabilities of the Screening Strategies That Perform Well Are Not Good

From the results in Figure 9 and Figure 12, it was observed that the standard variances of confirmed numbers obtained by using Seq-M and LL-NC are apparently larger. Therefore, although the overall effects are better, Seq-M and LL-NC cannot always maintain the best performance. This is a shortcoming of our proposed strategies, and we will continue to research the potential reasons for this phenomenon.

## 5. Conclusions

To improve the screening efficiency and avoid larger diffusions during the early stage of the epidemic, we propose an SSF to generate different strategies based on the trajectory network. According to the SSF, we propose using six types of screening strategies that include the identification of influential persons and the screening modes. To examine the efficiency of our proposed screening strategies, we built a scenario model that involves the general behaviors of people in normal life, the typical behaviors of people during the pandemic, and information spreading among individuals. In addition, the Monte Carlo method was used to conduct our experiments. Two types of scenarios were designed: the 1st is screening people after the first confirmed case appears, and the 2nd is screening people before the first confirmed case appears. The experimental results demonstrate that (1) our proposed screening strategies can be used to improve the efficiency of controlling disease diffusion, and (2) Seq-M (i.e., sequential screening by $M_{i}$ value of people in a screening period) is the most efficient in the 1st scenario, with LL-NC (i.e., long-line screening by $NC_{i}$ value of people in a screening period) being the most efficient in the 2nd scenario. Thus, after the first confirmed case appears, it is important to pay more attention to people who have larger contact numbers and contact frequencies with others, such as disease control officers, and we should screen them sequentially by their contact metrics. During regular screening, individuals who always contact different people should be preferentially concerned, such as travelers, and we should screen them according to their contact lines. Moreover, we found that the stabilities of Seq-M and LL-NC were not very comparable, and research on the potential reasons for this phenomenon and improvement of these screening strategies needs to be conducted in the future.

## Figures and Tables

**Table 1:** The parameters in the scenario model. Some parameters are designed as probability distributions, including the discrete uniform distribution (uniform_discr), Poisson distribution, and normal distribution.

No.	Parameters	Value	Remark
1	Family size	1000	
2	Population size	2890–3100	
3	Personnel density	23,885 people per square kilometer	The personnel density of a city in China.
4	Initial infected person	1	There is 1 infected person at the beginning of the simulation.
5	Initial warning/rumor spreaders	uniform_discr (10, 20)	10–20 people are warned at the beginning of the simulation.
6	Initial infected persons in a day	Poisson (1)	Before the control, 0–4 new initially infected people (whose distribution conforms to the Poisson distribution) may appear in the crowd every day.
7	New spreaders of warning/rumor in a day	Poisson (5)	After the control, 0–15 initial warning or rumor spreaders (whose distribution conforms to the Poisson distribution) may be generated in the crowd every day.
8	Detection reagents	adequate	Assumption.
9	Immunities of people	abs (normal (0.1, 0.7))	People’s immunities are in the range of 0.2–1.2, which conforms to the normal distribution, and the asymptomatic peoples’ immunities exceed 0.9.
10	Incubation duration	this.immunity * 10	People’s incubation durations are in the range of 2–12 days, which conform to the normal distribution, and the middle value is 7 days.
11	Infectious distance	abs (normal (1.2, 6))	The distances that can create infections are in the range of 2–12 m. People can be infected if the real distance is smaller than this parameter.
12	Infectious rate	One time per 1 min	People can diffuse the virus every minute if they have contacts.
13	Mortality	uniform (0.002, 0.003) * (confirmed time -infected time)	Mortality is related to the time when the infected person is confirmed. We assume that mortality is approximately 0.03–0.05.
14	Treatment duration	abs (normal (1, 10))	The treatment durations of confirmed people are in the range of 6–14 days, which conforms to the normal distribution.
15	Detection duration	abs (normal (0.2, 1))	The detection durations of people are in the range of 0–2 days, which conforms to the normal distribution, and the middle value is 1 day.
16	Close contact tracing rate	2 h	More details as follows.

* indicates the multiplication sign in the code language.

**Table 2:** Design of the experiments conducted in this paper.

Application Scenario	Application Strategy
/	No action
/	Only keeping social distance
After the first confirmed case appears (which is named the 1st scenario)	Keeping social distance and the random screening strategy
	Keeping social distance and the Seq-M
	Keeping social distance and the LL-M
	Keeping social distance and the Fam-M
	Keeping social distance and the Seq-NC
	Keeping social distance and the LL-NC
	Keeping social distance and the Fam-NC
Before the first confirmed case appears (which is named the 2nd scenario)	Keeping social distance and the random screening strategy
	Keeping social distance and the Seq-M
	Keeping social distance and the LL-M
	Keeping social distance and the Fam-M
	Keeping social distance and the Seq-NC
	Keeping social distance and the LL-NC
	Keeping social distance and the Fam-NC

## Data Availability

The data presented in this study are available on request from the corresponding author. The data are not publicly available due to privacy restrictions.
